# A Zebrafish Embryo Model to Screen Potential Therapeutic Compounds in *Sapindaceae* Poisoning

**DOI:** 10.3390/molecules29204954

**Published:** 2024-10-19

**Authors:** Clovis P. Wouters, Benjamin Klein, Nicholas Price, François Boemer, Marianne L. Voz, Dominique-Marie Votion

**Affiliations:** 1 Department of Functional Sciences, Faculty of veterinary Medicine, Pharmacology and Toxicology, Fundamental and Applied Research for Animals & Health (FARAH), University of Liège, 4000 Liege, Belgium; benjamin.klein@uliege.be (B.K.); dominique.votion@uliege.be (D.-M.V.); 2 Department of Biochemistry, University of Alberta, Edmonton, AB T6G 2R3, Canada; njprice@ualberta.ca; 3 Biochemical Genetics Laboratory, Human Genetics, CHU Sart Tilman, University of Liège, 4000 Liege, Belgium; f.boemer@chuliege.be; 4 Laboratory of Zebrafish Development and Disease Models (ZDDM), GIGA, University of Liège, Sart Tilman, 4000 Liege, Belgium; mvoz@uliege.be

**Keywords:** hypoglycin A, methylenecyclopropylacetate, methylenecyclopropylglycine, zebrafish larvae, Zebrabox^®^

## Abstract

Hypoglycin A (HGA) and methylenecyclopropylglycine (MCPrG) are protoxins produced by *Sapindaceae* plants, particularly *Acer pseudoplatanus*, and are responsible for causing atypical myopathy (AM) in equids. These protoxins metabolise into toxic compounds, such as methylenecyclopropylacetyl-CoA (MCPA-CoA), which alters energy metabolism and induces severe rhabdomyolysis. Currently, no specific treatment exists for this poisoning, in vitro models fail to reproduce HGA’s toxic effects on equine primary myoblasts, and mammalian models are impractical for large-scale drug screening. This study aimed to develop a zebrafish embryo model for screening therapeutic compounds against AM. Zebrafish embryos were exposed to various concentrations of HGA, MCPrG, and methylenecyclopropylacetate (MCPA) for 72 h. MCPrG did not induce toxicity, while HGA and MCPA showed median lethal concentration (LC50) values of 1.7 µM and 1 µM after 72 h, respectively. The highest levels of the conjugated metabolite MCPA–carnitine were detected 24 h after HGA exposure, and the acylcarnitines profile was highly increased 48 h post-exposure. Isovaleryl-/2- methylbutyrylcarnitine levels notably rose after 24 h, suggesting potential exposition biomarkers. Glycine and carnitine effectively reduced mortality, whereas riboflavin showed no protective effect. These findings suggest that the zebrafish embryo represents a valuable model for identifying therapeutic compounds for *Sapindaceae* poisoning.

## 1. Introduction

Invasive or newly encountered flora present an emerging risk of toxic exposure not only to grazing fauna but also to humans through both direct and indirect pathways, the latter via the consumption of animal-derived products such as milk and meat [1]. Hypoglycin A (HGA) and methylenecyclopropylglycine (MCPrG) are protoxins synthesised by plants of the *Sapindaceae* family such as lychee (*Litchi chinensis*), ackee (*Blighia sapida*) [2,3,4] and some maple trees notably (*Acer pseudoplatanus*) [2]. The ingestion of *A. pseudoplatanus* (sycamore maple) components, known for its association with emerging pasture-related toxicities, triggers atypical myopathy (AM) in equines. Toxicity is attributed to all components of the plant, including fruits, leaves, and seedlings [5,6,7,8]. Atypical myopathy is characterised as an acute rhabdomyolysis syndrome, triggered by the consumption of HGA and MCPrG, compounds found in the seeds and seedlings of *A. pseudoplatanus*. Upon ingestion, these protoxins undergo metabolic conversion into methylenecyclopropylacetyl-CoA (MCPA-CoA) and methylenecyclopropylformyl-CoA (MCPF-CoA), respectively. These metabolites exert their toxicological effects by the inhibition of the acyl-CoA dehydrogenases of short- and medium-chain, inhibiting multiple stages of β-oxidation, thus disrupting lipid metabolism. These inhibitions led to a strong increase in the corresponding acylcarnitines in blood and skeletal muscles [9,10]. Currently, supportive treatments such as the administration of vitamins and antioxidants have been shown to enhance the survival of horses affected by AM [11]. However, no specific treatment currently exists for this poisoning, leading to a lethality rate between 61 and 74% [12,13]. Thus, the development of an efficient drug screening model would be highly valuable to reduce the impact of the poisoning.

Since the discovery of toxins primarily associated with Jamaican vomiting sickness, various in vitro models [14] and in vivo models (e.g., rabbit, mice, rat) [15,16,17] have been utilised to characterise the toxicity of HGA and methylenecyclopropylacetate (MCPA). As part of AM investigations, the cytotoxicity of these toxins has also been studied using in vitro models such as cultured equine primary myoblasts [18,19,20]. Notably, these studies revealed that, while MCPA exhibited cytotoxic effects, HGA at concentrations of 1 mM and 10 mM did not induce cytotoxicity in equine primary myoblasts [19,20], and thus, does not induce the toxic effects observed in vivo. This failure to replicate the full pathophysiological impact of HGA in vitro highlights the need for an alternative model capable of better simulating the intoxication process. In addition, while mammalian models, such as mice and rats [15,16,17], can reproduce the toxicity observed in equines, they are not practical for large-scale pharmaceutical screening. In fact, ethical concerns, logistical challenges, and the high costs associated with maintaining and conducting experiments on large numbers of mammals render them unsuitable for high-throughput drug discovery [21]. This has resulted in a gap in models suitable for characterising and understanding the cytotoxicity of *A. pseudoplatanus* toxins.

Various models have been developed to address these challenges, including complex in vitro models [22], *Caenorhabditis elegans* [23], or zebrafish. Zebrafish are a powerful tool in toxicity assays, offering advantages like genetic similarity to humans, transparent embryos, and rapid development as well as easy to maintain, and allow for rapid, high-throughput screening [24,25,26]. These features facilitate the efficient, real-time observation of toxic effects across environmental and pharmaceutical studies. Their use in high-throughput screenings offers a cost-effective method for assessing compound toxicity, with direct exposure to the substances facilitated by the larvae’s permeability. This model has proven valuable in identifying organ-specific toxicities and mechanisms of action, enhancing our understanding of the potential human health impacts and contributing to safer chemical and therapeutic development [27,28]. Furthermore, zebrafish are a valuable tool in drug discovery, with applications in drug screening, target identification, pharmacology, and toxicology [29,30,31,32]. In the field of toxicology, zebrafish can be used to identify the molecules with protective effects against various toxins [32,33], making them an ideal model for evaluating potential therapeutic compounds against *Sapindaceae* poisoning.

This study aimed to longitudinally investigate the effects of HGA, MCPrG, and MCPA in zebrafish larvae and to develop a drug screening model for identifying therapeutic compounds.

## 2. Results

### 2.1. Zebrafish Embryo Acute Toxicity Test

Prior to conducting the zebrafish embryo acute toxicity tests (*z*FETs), a range finding test (RFT) was performed before the zFET, enabling the investigation of the suitable concentrations for the zFET and to assess the solubilisation and resorption of toxic compounds (i.e., HGA, MCPrG, and methelynecyclopropylacetate also known as MCPA) in solvents comprising E3 medium, 1% dimethyl sulfoxide (DMSO) and 1% ethanol. No difference was observed regarding the sub-lethal and lethal effects when toxic compounds (i.e., HGA, MCPrG, and MCPA) were dissolved 1% DMSO or 1% ethanol in comparison versus in E3 medium. Therefore, the absorption of toxic molecules was supposed to be similar between solvents leading to solubilisation in E3 medium for the zFET. Details regarding the tested concentrations of the RFT are displayed in (Supplementary Table S1).

**Figure 1:**
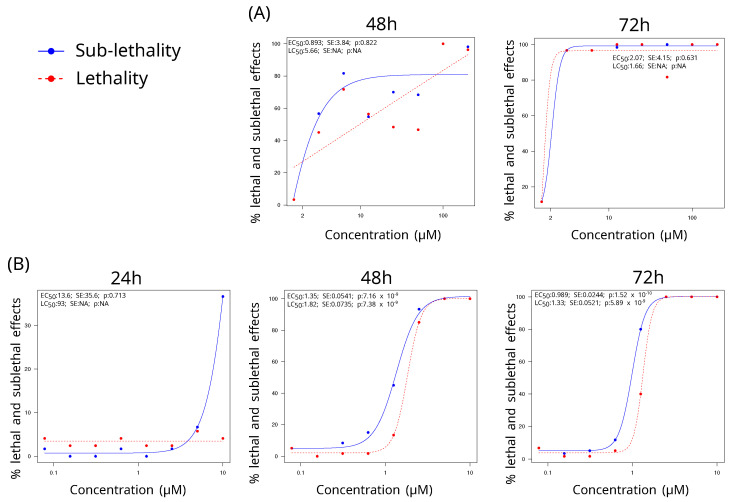
Lethality and sub-lethal effects of zebrafish embryos exposed to various concentrations of *A. pseudoplatanus* toxins, hypoglycin A, and methelynecyclopropylacetate over 72 h. The experiment was conducted to determine the median lethal (LC50) and median sub-lethal (EC50) concentration for hypoglycin A (**A**) and methelynecyclopropylacetate (**B**) at 24, 48, and 72 h post-exposure. Data were obtained from three independent replicates with 20 embryos per concentration per replicate. The LC50 and EC50 values were calculated using a four-parameter log-logistic model implemented with the drc R package, along with the associated standard error and the p-value. The sample size was 60 individuals per concentration (3 trials × 20 individuals), and the statistical test was a logistic regression with four parameters.

The zFET was initiated to expose larvae to toxic compounds (i.e., HGA, MCPrG, and MCPA) at 24 hpf. The median lethal concentration (LC50) and the median effective concentration (EC50) were determined for the three compounds at different time points, specifically at 24, 48, and 72 h post-exposure (Figure 1). Despite the increase in MCPrG concentrations up to 5000 μM, no sub-lethal or lethal effects were observed throughout the RFT, even extending to 96 h post-exposure. This absence of observable effects precluded the application of the zFET assay for MCPrG. Similarly, no effects were observed for HGA intoxication after 24 h. Therefore, it was not possible to determine LC50 and the EC50 values MCPrG and HGA after 24 h post-exposure.

Dose-dependent mortality was observed for both HGA and MCPA with cumulative mortality rates increasing over time (Figure 1). Observations revealed a progressive increase in both the incidence and severity of sub-lethal effects throughout the duration of the toxic exposure. The lethal and sub-lethal effects were similar between both HGA and MCPA. Notably, no adverse effect was observed at 24 h post-exposure with HGA. In contrast, significant effects manifested within a few hours following a high concentration of MCPA in the RFT. The lethal effects as described in OECD guideline included egg coagulation and lack of heartbeat, with the egg coagulation being exclusively associated with MCPA, given the fact that larvae died at 72 hpf with HGA (i.e., 48 h post-exposure). The primary lethal point was lack of heartbeat. Among the sub-lethal effects, bradycardia were predominantly observed, followed by pericardial oedema, and cardiac arrhythmia, in decreasing order of frequency.

Thanks to a the four-parameter log-logistic function, the LC50 and EC50 values were determined. HGA-induced toxicity appeared after 48 h, primarily characterised by a bradycardia and increased mortality. The severity of these effects remained relatively consistent, fluctuating between 40% and 80% for HGA concentrations ranging from 3.125 to 50 μM. Notably, the effects approached 100% lethality at concentrations of 100 and 200 μM HGA (Figure 1 panel A). After 72 h, the estimated EC50 was 2.07 μM, while the estimated LC50 was 1.66 μM, although this finding was not statistically significant (Figure 1 panel A). In comparison, exposure to MCPA resulted in sub-lethal effects in approximately 30% of the specimens at 24 h post-exposure with a concentration of 10 μM. By 48 h, the EC50 and LC50 values were calculated to be 1.35 and 1.82 μM, respectively, and by 72 h, these values decreased to 0.989 and 1.33 μM, respectively (Figure 1 panel B). Minimal to no effects were observed at concentrations below 0.3125 μM.

### 2.2. Toxin, Toxic Metabolite Dosage, and Acylcarnitines Profile

**Figure 2:**
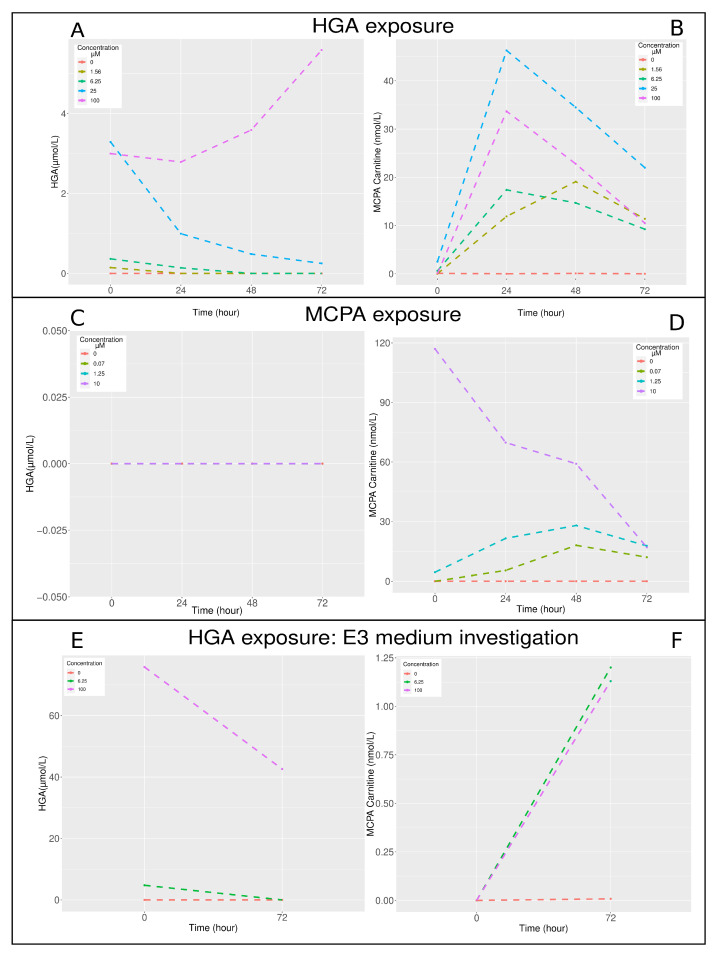
Time course of HGA and MCPA–carnitine concentrations in zebrafish larvae (24 hpf) after intoxication with HGA and MCPA. Hypoglycin A (μmol/L) and MCPA–carnitine (nmol/L) concentrations in the zebrafish larvae whole body after intoxication with hypoglycin A in (**A**,**B**), respectively, after MCPA exposure in (**C**,**D**), respectively. Hypoglycin A (μmol/L) and MCPA–carnitine (nmol/L) concentrations in the E3 medium surrounding the zebrafish larvae after intoxication with HGA in (**E**,**F**), respectively. The sample size was 20 individuals per concentration (1 trial × 20 individuals). HGA: hypoglycin A; MCPA: methylenecyclopropylacetate.

Longitudinal monitoring of HGA, MCPA–carnitine, and acylcarnitines profile was undertaken to elucidate the kinetics of intoxication (Figure 2). After HGA exposure, the concentration of HGA in zebrafish larvae exhibited a decline, with the exception observed at the 100 μM HGA exposure level (Figure 2 panel A). The protoxin HGA was metabolised into MCPA in zebrafish larvae. The most pronounced elevations in MCPA–carnitine concentrations were observed after 24 h at HGA concentrations exceeding 6 μM, and after 48 h for a 1.56 μM HGA concentration (Figure 2 panel B). Following MCPA exposure, HGA was undetectable (Figure 2 panel C). The peak levels of MCPA–carnitine were recorded after 48 h for exposures to 1.25 and 0.07 μM MCPA. In contrast, at a 10 μM MCPA concentration, the MCPA–carnitine level was exceptionally high initially at 0 h and exhibited a decrease over the course of 72 h (Figure 2 panel D).The concentration of HGA in the E3 medium reduced over time and was proportional to the initial HGA concentrations exposure (Figure 2 panel E), whereas the MCPA–carnitine levels in the E3 medium increased over time and were proportional to the initial HGA concentrations exposure (Figure 2 panel F). However, similar MCPA–carnitine concentrations were measured for exposures to 6.25 and 100 μM HGA.

**Figure 3:**
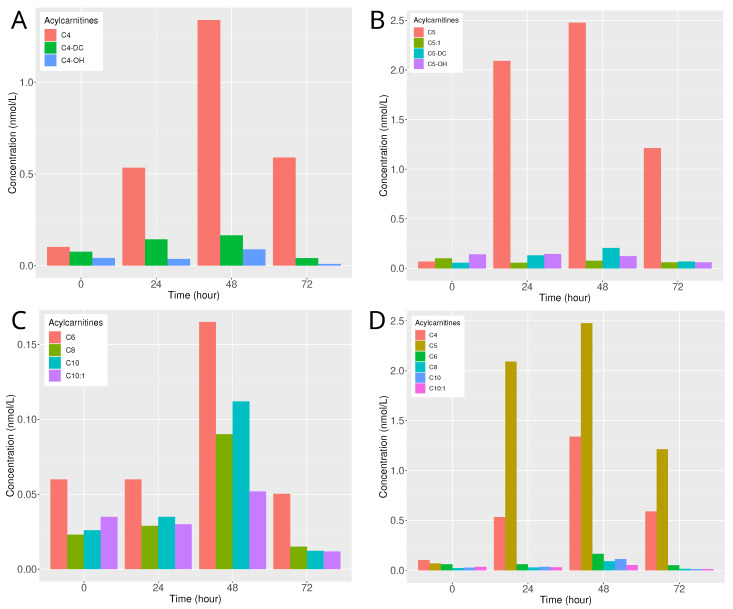
Time course of acylcarnitines concentrations in the whole body of zebrafish larvae after exposure with 100 μM of hypoglycin A. Concentrations of four-carbon chain length in (**A**), five-carbon chain length acylcarnitines in (**B**), even-numbered acylcarnitines in (**C**) and short- and medium chain acylcarnitines in (**D**). The sample size was 20 individuals per concentration (1 trial × 20 individuals). DC: dicarboxylic.

The goal of this analysis was to evaluate the kinetics of the acylcarnitines profile after HGA exposure. The kinetics of the acylcarnitines profile were analysed following the exposure of zebrafish larvae to 100 μM HGA. Notably, a significant increase in acylcarnitines with a four-carbon chain length was observed after 48 h, particularly for butyryl-/isobutyrylcarnitine (C4), while the magnitude of the increase was more moderate for hydroxy butyryl-/isobutyrylcarnitine (C4-OH) and succinyl-/methylmalonylcarnitine (C4-DC), (Figure 3 panel A). Interestingly, at 24 and 72 h post-exposure, butyrylcarnitine levels remained relatively elevated, whereas levels of C4-OH and C4-DC experienced a marked decrease after 72 h (Figure 3 panel A). The kinetics of isovaleryl-/2-methylbutyrylcarnitine (C5) showed a pronounced increase 24 h after exposure, maintaining elevated levels at 48 h (Figure 3 panel B). Conversely, other five-carbon length acylcarnitines, such as tiglylcarnitine (C5:1), hydroxy isovalerylcarnitine (C5-OH), and glutarylcarnitine (C5-DC), remained close to the baseline levels observed prior to exposure (Figure 3 panel B). Among the even-numbered acylcarnitines, hexanoylcarnitine (C6), octanoylcarnitine (C8), and decanoylcarnitine (C10) levels increased after 48 h (Figure 3 panel C). Among the short- and medium-chain acylcarnitines, C4 and C5 exhibited the most significant increases (Figure 3 panel D).

### 2.3. Short-Term Toxicity Test and Locomotor Behaviour Assessment

**Figure 4:**
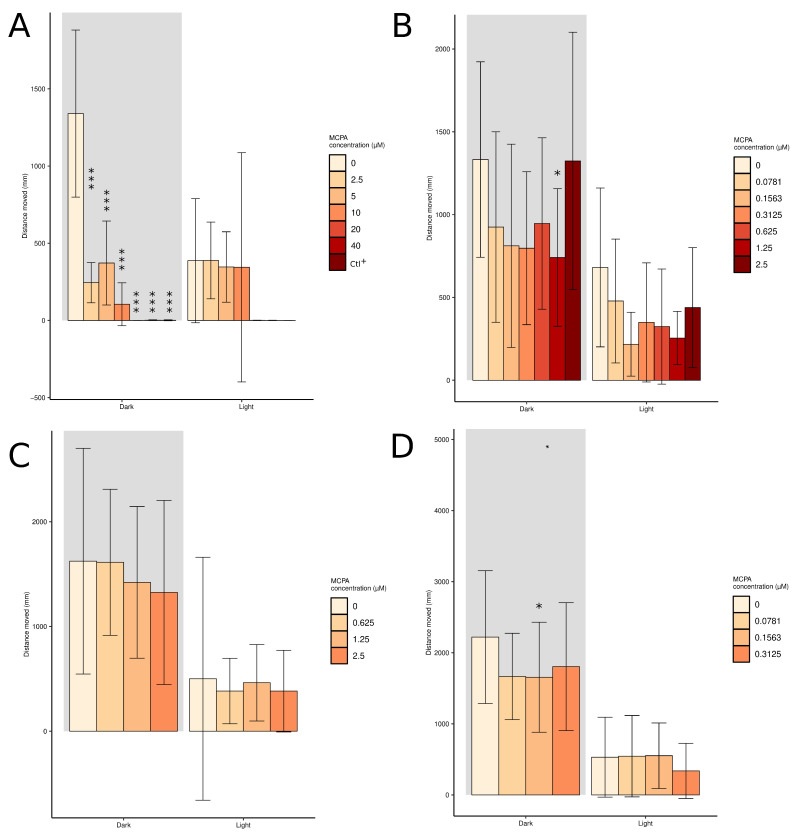
Total swimming distance in four-day post-fertilisation zebrafish larvae exposed to various concentrations of methylenecyclopropylacetate from 72 to 96 h post-fertilisation. The sample size was 12 individuals per concentration in (**A**,**B**) and 24 individuals per concentration in (**C**,**D**). (*: *p* < 0.05, ***: *p* < 0.001 according to one-way ANOVA and Dunnett’s post hoc test). MCPA: methylenecyclopropylacetate; Ctl^+^: 3,4-dichloroaniline.

Total swimming distance was investigated to detect short-term toxicity in larvae exposed to various concentrations of MCPA (Figure 4). Twenty minute exposure of high concentrations of MCPA did not reduce the swimming distance during dark photocycle compared to the control (Supplemental Figure S2 panel A). However, twenty-four hours of exposure of high concentrations of MCPA significantly reduced the swimming distance during dark photocycle (Figure 4 panel A). The larvae exposed to 3,4 dichloroaniline (positive control), 40 and 20 µM MCPA for 24 h were dead (Figure 4 panel A). Twenty minutes to two hours of exposure to low concentrations of MCPA did not reduce the swimming distance during the dark photocycle (Supplemental Figure S2 panels B–D). Twenty-four hours of exposure to 1.25 µM and 0.15 µM of MCPA significantly reduced the swimming distance during dark photocycle compared to the control (Figure 4 panel B,D).

### 2.4. Impact of Riboflavin, Carnitine, and Glycine on Zebrafish Larval Mortality under MCPA and HGA Exposure

**Figure 5:**
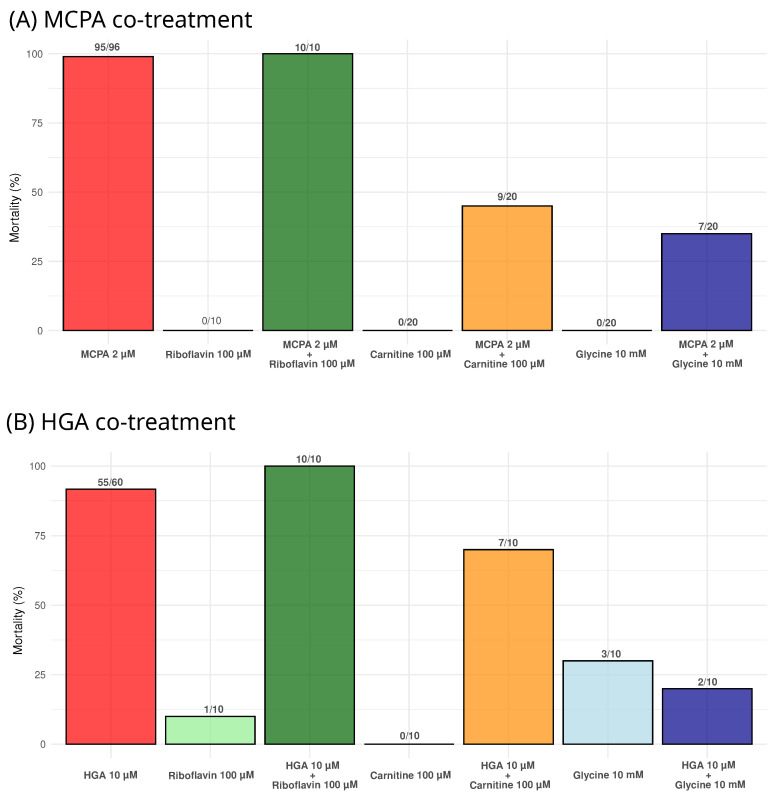
Assessment of riboflavin, carnitine, and glycine co-treatment effects on the mortality of zebrafish larvae in the presence of MCPA (in (**A**)) or HGA (in (**B**)) after 72 h of exposure. The absolute number of larval deaths is indicated above each bar. MCPA: methylenecyclopropylacetate; HGA: hypoglycin A.

The protective effects of riboflavin, carnitine, and glycine were evaluated after 72 h of co-exposure with MCPA or HGA (Figure 5). Positive controls consisted of larvae exposed to toxic compounds only, while negative controls consisted of larvae in E3 medium and with the test molecules alone.

Riboflavin did not show a protective effect when co-treated with either MCPA or HGA, while carnitine and glycine decreased mortality under both conditions (Figure 5). For carnitine, all surviving larvae exhibited sub-lethal effects (bradycardia or cardiac oedema) with MCPA (11/11), whereas one out of three did so with HGA. Glycine demonstrated greater protective efficacy, with fewer surviving larvae showing sub-lethal effects with MCPA (6/13), and none with HGA (0/8).

Most of the positive controls succumbed within 72 h. Those that survived displayed significant sub-lethal effects and were likely to die shortly thereafter. Among the negative controls, three out of ten larvae in the glycine group and one in the riboflavin group died after 72 h for reasons that remain unknown.

## 3. Discussion

In this study, we developed a zebrafish embryo model to assess the toxic effects of protoxins derived from *A. pseudoplatanus* and to screen potential therapeutic compounds for *Sapindaceae* poisoning. Zebrafish embryos at 24 hpf were exposed to various concentrations of HGA, MCPrG, and MCPA. The longitudinal design of the study allowed us to monitor both lethal and sub-lethal effects, including the quantification of HGA and MCPA–carnitine, as well as acylcarnitines profile. Behavioural responses were also recorded, providing an additional dimension of toxicological insight. Lastly, the potential protective effect of riboflavin, carnitine, and glycine, three supportive treatments used in AM, was evaluated using this model.

Our findings confirm that HGA and MCPA induce significant toxic effects in zebrafish larvae, consistent with their known role in inhibiting fatty acid metabolism [16,34]. Apart from death, two key sub-lethal toxicity endpoints were identified: diminished cardiac rate and cardiac oedema. Under normal conditions, zebrafish larvae exhibit heart rates between 120 and 180 beats per minute [35], but exposure to HGA and MCPA reduced heart rates to below 80 beats per minute. Cardiac oedema was also commonly observed, a condition known to be induced by other toxic compounds [36,37]. Larvae with cardiac oedema typically exhibited reduced heart rates. These findings align with reports of cardiac signs occasionally seen in horses affected by AM [38]. Additional signs, such as tremors, shivering, and dorsal axis curvature [39], were occasionally noted but were not classified as sub-lethal effects, as they were also sporadically observed in control groups.

Previous studies in mammals have demonstrated that HGA inhibits acyl-CoA dehydrogenases [14,40,41], leading to the accumulation of toxic metabolites and the disruption of energy production, which can be monitored through the acylcarnitines profile [9,42]. In our study, the kinetics of HGA, MCPA–carnitine, and the acylcarnitines profile in the zebrafish whole body were notably similar to those observed in human serum [43], supporting several key insights. Firstly, following HGA exposure, the highest concentrations of HGA in zebrafish larvae were detected shortly after exposure, aligning with reports of elevated HGA serum levels in humans one hour after ackee ingestion. Interestingly, larvae exposed to the highest concentration (100 µM) displayed an unexpected increase in internal HGA levels over time, while lower concentrations showed the expected decline due to metabolisation into MCPA-CoA. Although this discrepancy remains unexplained, it suggests that potential metabolic or absorption dynamics from the media that require further investigation. The concurrent decrease in HGA concentration in the E3 medium supports ongoing metabolism or uptake by the larvae. While technical factors, such as the loss of the chorion [44], may influence dosage measurements, these findings provide new insights into HGA kinetics in zebrafish larvae. Secondly, the metabolism of HGA into MCPA–carnitine started within the first 24 h, leading to high MCPA–carnitine levels observed 24 h after HGA exposure, particularly at concentrations above 30 nmol/L. These levels decreased over time at higher HGA concentrations exposure (100 and 24 µM), while remaining steady between 20 and 10 nmol/L for lower exposure concentrations (6.25 and 1.58 µM). The increase in MCPA–carnitine levels coincided with a decrease in HGA concentrations in both the larvae and the E3 medium, mirroring human studies where HGA serum concentrations similarly declined 24 h after ackee fruit consumption [43]. Thirdly, the acylcarnitines profile revealed significant metabolic disruptions, especially in butyryl-/isobutyrylcarnitine (C4) and isovaleryl-/2-methylbutyrylcarnitine (C5), which peaked 48 h post-HGA exposure. Among short- and medium-chain acylcarnitines, C5 showed a more significant increase and may serve as a more predictive biomarker than C4 of HGA exposition. While C4 levels remained stable at 24 and 72 h post-exposure, C5 levels exhibited more dynamic fluctuations during this period. Although differences in dosing methods and sample matrices exist, the overall kinetic patterns of HGA, MCPA–carnitine, and acylcarnitine profiles in zebrafish larvae are closely similar to those seen in human serum.

Methylenecyclopropylglycine (MCPrG) did not induce toxic effects in zebrafish larvae, even at a concentration of 5 mM after 72 h of exposure. Previous studies in mammals, including mice and rats, demonstrated the toxic effects of MCPrG [16,17], with the primary difference being the route of administration (intraperitoneal or oral versus immersion in medium). In our study, absorption was assessed by comparing the dissolution of toxic molecules in E3 medium, 1% dimethyl sulfoxide (DMSO), and 1% ethanol, with no significant differences in mortality among the solvents. The chorion may have played a protective role, as it can limit the passage of molecules based on size and polarity [45]. While the zebrafish chorion is generally permeable to small molecules under 3000 Da [45,46,47,48,49], whether MCPrG crosses it remains unconfirmed. Most embryos lost their chorion between 48 and 72 hpf, which might have influenced absorption. However, no differences in mortality were observed with other toxins (HGA, MCPA) between larvae with or without chorion during the method development. Metabolism of both *A. pseudoplatanus* toxins relies on branched-chain amino acid aminotransferase and the branched-chain α-ketoacid dehydrogenase complex [16], but it is unknown whether metabolic differences exist between zebrafish and humans. Quantifying MCPF–carnitine in both medium and whole body could provide further insights. Additionally, the 72-h observation period may have been insufficient, as the toxic effects of HGA became evident only after 48 h, fully manifesting at 72 h.

Behavioural analysis showed that high concentrations of MCPA significantly reduced the swimming distance during the dark photocycle, a response not immediately observed after exposure. Additionally, high MCPA concentrations altered movement patterns and induced thigmotaxis, the tendency of an animal to stay in contact with a vertical surface [50], a behaviour typically associated with anxiety [51]. This effect was prominent after 24 h of exposure (Figure 4 panel A; Figure S3). In contrast, lower concentrations of MCPA did not significantly impact swimming distance or behaviour, suggesting a concentration-dependent effect of MCPA on zebrafish larvae.

One limitation of this study is the use of 24 hpf zebrafish larvae for toxin exposure, instead of the recommended 0–3 hpf stage as outlined by OECD guidelines [52]. Initially, we conducted experiments using 0.5–3 hpf embryos, but encountered significant challenges in consistently identifying fertilised eggs. Approximately 5% of the eggs were unfertilised, introducing variability across experimental conditions and compromising reproducibility, particularly with large sample sizes of around 2000 eggs per experiment. Additionally, manual observation under the microscope is time-consuming and challenging, hindering the ability to ensure the precise timing of exposure for each egg, which introduced further variability. To improve reproducibility, we opted to begin exposures at 24 hpf, when fertilised and unfertilised eggs could be more easily distinguished using fluorescence from methylene blue in the E3 medium. Although this modification may bypass potential toxic effects during the first 24 h, our primary goal was to ensure reliable data for therapeutic compound screening, and 24 hpf embryos better reflect the physiology of developed animals affected by *Sapindaceae* poisoning. Furthermore, while this adaptation limits our ability to assess certain endpoints (e.g., coagulation, somite formation, tail detachment), our control larvae under identical conditions displayed no significant sub-lethal or lethal effects, suggesting that the use of 24 hpf embryos remains suitable for our objectives. However, future studies should consider including earlier exposure stages for a more comprehensive toxicological profile.

Another limitation of this study is the lack of replication for the quantification of HGA, MCPA–carnitine, and acylcarnitines profile. Therefore, these results should be interpreted with caution. To increase robustness, the experiments would need to be repeated at least three times to generate biological replicates. However, as the primary focus of the study was not the detailed quantification of these profiles but rather the identification of therapeutic compounds, we chose not to perform multiple replicates. We prioritised the screening of potential therapeutic agents, which we believe will have a greater positive impact on treating poisoned animals.

The protective effects of three molecules (riboflavin, glycine, and carnitine), known for their potential protective roles in AM, were evaluated in this study [12,53,54]. Riboflavin did not demonstrate any protective effect in our experiments, which aligns with a previous in vitro study where co-treatment with riboflavin did not protect primary myoblasts exposed to MCPA [20]. However, a protective effect of riboflavin on liver mitochondria in rats was observed when administered prior to HGA exposure [55]. Therefore, the protective potential of riboflavin in intoxicated mammals remains unclear.

In contrast, carnitine and glycine exhibited protective effects in zebrafish larvae exposed to both HGA and MCPA (Figure 5). Both molecules are capable of binding MCPA to form MCPA–carnitine or MCPA–glycine complexes, facilitating the elimination of MCPA [34,43,54]. These findings are consistent with a previous in vitro study, where co-treatment with carnitine and glycine protected primary myoblasts exposed to MCPA [20]. Although most of the surviving larvae displayed sub-lethal effects, the delay in lethality is notable in the context of *Sapindaceae* poisoning, as horses that survive AM typically recover fully. These preliminary results support the recommendation to supplement with carnitine and glycine during *Sapindaceae* poisoning [54]. However, further studies are needed to validate these protective effects in other animal models and to explore the underlying mechanisms in more detail.

## 4. Materials and Methods

### 4.1. Materials

The protoxins HGA and MCPrG were obtained from Toronto Research Chemicals (North York, ON, Canada), while MCPA was purchased from Sigma-Aldrich (Overijse, Belgium) (Table 1).

### 4.2. Zebrafish Husbandry

Zebrafish (*Danio rerio*) of the AB wildtype strain were maintained in a Techniplast rearing system at 28 °C under a 14 h/10 h light/dark photocycle within the zebrafish facility at GIGA-Research, University of Liège. Following breeding, fertilised eggs were maintained in E3 medium (5 mM NaCl, 0.17 mM KCl, 0.33 mM CaCl_2_, 0.33 mM MgSO_4_) in Petri dishes at 28 °C until 24 h post-fertilisation (hpf). All procedures adhered to animal protection guidelines and were approved by the Animal Ethics Committee of the University of Liège.

### 4.3. Zebrafish Embryo Acute Toxicity Test

A RFT was initially conducted using ten individuals in a single experiment to define the effective concentrations of HGA, MCPA, and MPCrG (Supplementary Table S1). Ten eggs per chemical concentration, sixteen eggs for the negative control (E3), and twenty eggs for the positive control (4 mg/L of 3,4 dichloroaniline, Sigma Aldrich, Overijse, Belgium) with one egg per well in 100 μL of solution were exposed in 96-well plates from approximately two-cell stage to 96 hpf without renewing the drug solutions. To assess whether the absorption of HGA, MCPA, and MCPrG was enhanced, 1% ethanol or 1% DMSO was added to the test solutions containing these toxic compounds.

Zebrafish embryo acute toxicity tests (*z*FETs) were performed in triplicate according to the OECD test guidelines [52] with a major modification: the eggs were intoxicated 24 h after breeding. Twenty fertilised eggs per concentration and four eggs for the negative control (E3) were exposed from approximately 24 hpf until 96 hpf with one egg per well in 24 well-plates (2 mL per well) or in 96 well-plates (120 μL per well) for MCPA and HGA, respectively. To avoid evaporation, all the plates were closed with an adhesive seal (VWR, Leuven, Belgium) and the edge wells of 96-well plates were filled with 300 μL of E3. In addition, a container filled with water was put in the incubator. Embryos were monitored every day from 24 until 96 hpf. Four apical points were monitored for mortality, as described in the OECD test guidelines: (i) coagulation; (ii) lack of somite formation; (iii) lack of detachment of the tail; and (iv) lack of heartbeat. Sub-lethality was characterised by slow heartbeat (i.e., less than 60 beats per minute) and cardiac oedema. Both mortality and sub-lethal effects enabled one to determine the LC50 and the EC50, respectively. Each exposure assay was always observed by the same observers (i.e., C.P.W. or N.P.) all along the experiment, avoiding inconsistent monitoring.

### 4.4. Acylcarnitines Profile

Individual larvae were initially isolated in separate wells and then pooled into groups of twenty per concentration, including negative controls (E3), and sampled at 0, 24, 48, and 72 h post-exposure. This approach not only mitigates intra-individual variability but also enables the analysis to remain within the quantification limits of the assays performed via liquid chromatography–mass spectrometry (LC-MS). This experiment was performed in one replicate. The larval stage occurs between 48 and 96 hpf, when it loses their chorion. The solution containing protoxins or toxic compounds was applied once without renewing solution all along the exposure. For the sampling collection, E3 medium was removed and two drops of tricaine methanesulfonate (MS-222, Sigma-Aldrich, 400 mg/L) were used for sacrifice. Tricaine methanesulfonate was removed and 200 μL of methanol 50% was added. Embryos/larvae were crushed using 10 back and forth processes through a 26 G and a 22 G needles. Samples were centrifuged at 2660 rcf. The supernatant was put at −20 °C for the LC-MS analysis. Hypoglycin A was quantified on the supernatant using a validated methodology based on an aTRAQ kit for the amino acid analysis of physiological fluids (Sciex, Framingham, MA, USA), as mentioned in Boemer et al. [56]. MCPA–carnitine and acylcarnitines profile concentrations in serum were determined by tandem mass spectrometry (MS/MS), as described previously [9].

### 4.5. Short-Term Toxicity Test and Locomotor Behaviour Assessment

The behaviours of individual exposed and control larvae were analysed using the ZebraBox^®^ recording chamber (Viewpoint, Lyon, France). After breeding, eggs were collected into E3 medium and maintained at 28 °C. On the following day, unfertilised eggs were removed and the E3 medium was changed daily. At 72 hpf, larvae were exposed to low or high MCPA concentrations, in 96-well plates (one larva and 120 μL per well). After exposure, the larvae were placed on the recording platform for tracking locomotor behaviour using the Zebrabox^®^ (Viewpoint, Lyon, France). The ZebraLab software (Viewpoint Life Sciences, Lyon, France) was used to program the light cycle protocol, which lasted one hour with 20 min warm-up followed by two cycles of a 10 min dark/light photocycle. Measurements were taken 20 min post-exposure and at one, two, three, and 24 h post-exposure. Twelve or twenty-four larvae per concentration were monitored. This experiment was performed in one replicate.

Zebrafish larvae react to an instantaneous shift from 100% to 0% illumination by markedly increasing their locomotor activity. Toxic compounds can significantly reduce this response, causing notably decreased activity during dark periods as compared to control. The assays were performed in the early afternoon between 12:00 and 15:00, as larvae tend to respond to illumination changes with the least variance during that time of day [57]. Locomotor data were aggregated in 600-second bins for each individual, and separated into nine locomotor activity variables as published by Pohl and collaborators [58].

### 4.6. Impact of Riboflavin, Carnitine, and Glycine on Zebrafish Larval Mortality under MCPA and HGA Exposure

The experimental conditions were consistent with those previously described in the zFET protocol. Experiments were conducted in biological triplicate in 24-well or 96-well plates with final volumes of 2 mL for MCPA and 200 µL for the HGA assessment, respectively. Each plate included at least two larvae either exposed to the test molecules alone or to the test molecules combined with the toxic compounds, multiple larvae exposed only to the toxic compounds as positive controls, and several larvae maintained in E3 medium as negative controls. Multiple plates were utilised for the experiments. Riboflavin, glycine, and carnitine (CAS numbers: 83-88-5, 56-40-6, and 541-15-1, respectively) were obtained from Sigma-Aldrich (Overijse, Belgium) and added simultaneously with the toxic compounds, 2 µM of MCPA or 10 µM of HGA. The final concentration in the well for riboflavin, glycine, and carnitine was 100 µM, 10 mM, and 100 µM, respectively. Lethal and sub-lethal effects were observed every 24 h, as previously described.

### 4.7. Statistical Analysis

All data were exported and analysed using R (version 4.3.1 2023-06-16) [59] with the RStudio interface [60]. For the zFET, lethal and sub-lethal concentrations were calculated using the four-parameter log-logistic function using the drc R package [61]. For the dosage of HGA, MCPA–carnitine and acylcarnitines profile, plots were performed using the ggplot2 R package [62].

For locomotor analysis, data were analysed by adapting the R script published by Pohl and collaborators [58]. Briefly, individual total swimming distance (defined as smldist + lardist) during 10 min and light and dark periods were summarised. Control and treatment mean ± standard deviation were then plotted using the ggplot2 package [62]. Locomotor activity data (i.e., total swimming distance during dark conditions) were summarised for each individual embryo–larvae replicate and analysed using one-way analysis of variance (ANOVA) followed by normal distribution evaluation. Significant treatment effects were assessed by Dunnett’s post hoc test using the multcomp R package [63]. Treatment groups with *p*-values of <0.05 (*), <0.01 (**), and <0.001 (***) were considered as significantly deviating from the control mean.

## 5. Conclusions

In summary, we developed a zebrafish embryo model to assess the toxic effects of *A. pseudoplatanus* protoxins, HGA and MCPrG, as well as the toxic metabolite MCPA and to screen potential therapeutic compounds for *Sapindaceae* poisoning. Evaluations were conducted every 24 h over a 72 h period. Methylenecyclopropylglycine did not induce any toxic effects in zebrafish larvae, while HGA and its toxic metabolite, MCPA, exhibited significant toxic effects, allowing their LC50 and EC50 values to be determined.

Notably, the highest levels of MCPA–carnitine were detected 24 h after HGA exposure. The acylcarnitines profile, specifically C4, C6, C8, and C10, showed marked increases after 48 h. In contrast, C5 demonstrated a pronounced increase 24 h after exposure and maintained elevated levels at 48 h, confirming its potential as an early exposure biomarker in *Sapindaceae* poisoning [64].

Additionally, zebrafish larvae exposed to MCPA or HGA were utilised to screen the potential protective effects of molecules against AM. Among them, glycine and carnitine were effective in reducing mortality induced by MCPA and HGA, whereas riboflavin was not. This study introduces a novel model using zebrafish larvae and opens new perspectives for screening preventative or therapeutic compounds to mitigate AM-induced mortality.

## Figures and Tables

**Table 1:** List of tested molecules.

Supplier Name ^1^	Chemical Structure ^2^	Supplier (Cas Number)	Purity
(S)-Hypoglycin A	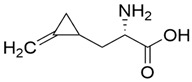	Toronto Research Chemicals (156-56-9)	85%
α-(Methylenecyclopropyl)glycine (mixture of diastereomers)	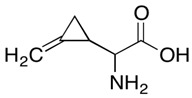	Toronto Research Chemicals (2517-07-9)	97%
(RS)-(Methylenecyclopropyl)acetic acid	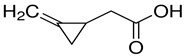	Sigma-Aldrich (1073-00-3)	≥95%

^1^ The molecules were abbreviated as HGA, MCPrG, and MCPA, respectively. ^2^ Chemical structures were taken from the suppliers’ respective website www.trc-canada.com; www.sigmaaldrich.com; all accessed on 4 July 2023.

## Data Availability

Data are contained within the article and Supplementary Materials.

## Supplementary Materials

The following supporting information can be downloaded at: https://www.mdpi.com/article/10.3390/molecules29204954/s1, Figure S1: Picture of a 96-hour post-fertilization zebrafish larva of displaying a cardiac oedema after toxin exposure; Figure S2: Total swimming distance in four-day post-fertilization zebrafish larvae exposed to various concentrations of methylenecyclopropylacetate; Figure S3: Schematic of zebrafish movement during a 10 min dark and light photocycle; Table S1: List of the tested concentrations during the experiments.

## Abbreviations

The following abbreviations are used in this manuscript:

AM	Atypical myopathy
C4	Butyryl-/isobutyrylcarnitine
C4-OH	Hydroxy butyryl-/isobutyrylcarnitine
C4-DC	Succinyl-/methylmalonylcarnitine
C5	Isovaleryl-/2-methylbutyrylcarnitine
C5:1	Tiglycarnitine
C5-OH	Hydroxy isovalerylcarnitine
C5-DC	Glutarylcarnitine
C6	Hexanoylcarnitine
C8	Octanoylcarnitine
C10	Decanoylcarnitine
DMSO	Dimethyl sulphoxide
N EC50	Median effective concentration
HGA	Hypoglycin A
LC50	Median lethal concentration
LC-MS	Liquid chromatography–mass spectrometry
MCPA	Methylenecyclopropylacetate
MCPA-CoA	Methylenecyclopropylacetyl-CoA
MCPrG	Methylenecyclopropylglycine
MS/MS	Tandem mass spectrometry
OECD	Organisation for Economic Co-operation and Development
RFT	Range finding test
zFET	Zebrafish embryo toxicity
