# Supplementary material for: A Zebrafish Embryo Model to Screen Potential Therapeutic Compounds in Sapindaceae Poisoning
Source: Molecules. 2024 Oct 19;29(20):4954. doi: 10.3390/molecules29204954 (PMC11510690; doi:10.3390/molecules29204954)
Supplement: Supplementary file 1 [file molecules-29-04954-s001.zip › molecules-3156444-supplementary.pdf]

SUPPLEMENTARY MATERIALS

A Zebrafish Zebrafish Embryo Model To Screen Potential Therapeutic Compounds in *Sapindaceae* Poisoning

Clovis P. Wouters[1*], Benjamin Klein[1], Nicholas Price[2], François Boemer[3], Marianne L. Voz[4] and Dominique-Marie Votion[3]

1 Department of Functional Sciences, Faculty of Veterinary Medicine, Pharmacology and Toxicology, Fundamental and Applied Research for Animals & Heathl (FARAH), University of Liège, 4000 Liège, Belgium;
2 Department of Biochemistry, University of Alberta, Edmonton, Alberta, Canada;
3 Biochemical Genetics Laboratory, Human Genetics, CHU Sart Tilman, University of Liège, 4000 Liège, Belgium;
4 Laboratory of Zebrafish Development and Disease Models (ZDDM), GIGA, University of Liège, Sart Tilman, 4000 Liège,

* Correspondence: clovis.wouters@uliege.be

Table of content

Supplementary Figure S1: ……………………………………………………………………..……S-2
Supplementary Figure S2: ……………………………………………………………………..……S-2
Supplementary Figure S3: ……………………………………………………………………..……S-3
Supplementary Table S1: ……………………………………………………………..…..……S-3

SUPPLEMENTARY Figure

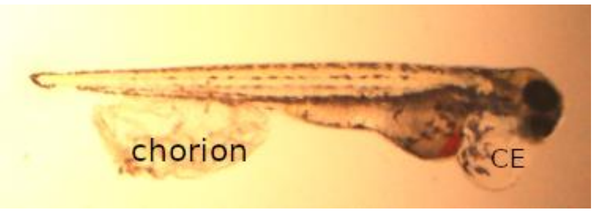

Figure S1. Picture of a 96-hour post-fertilization zebrafish larva of displaying a cardiac oedema after toxin exposure. CE: cardiac edema

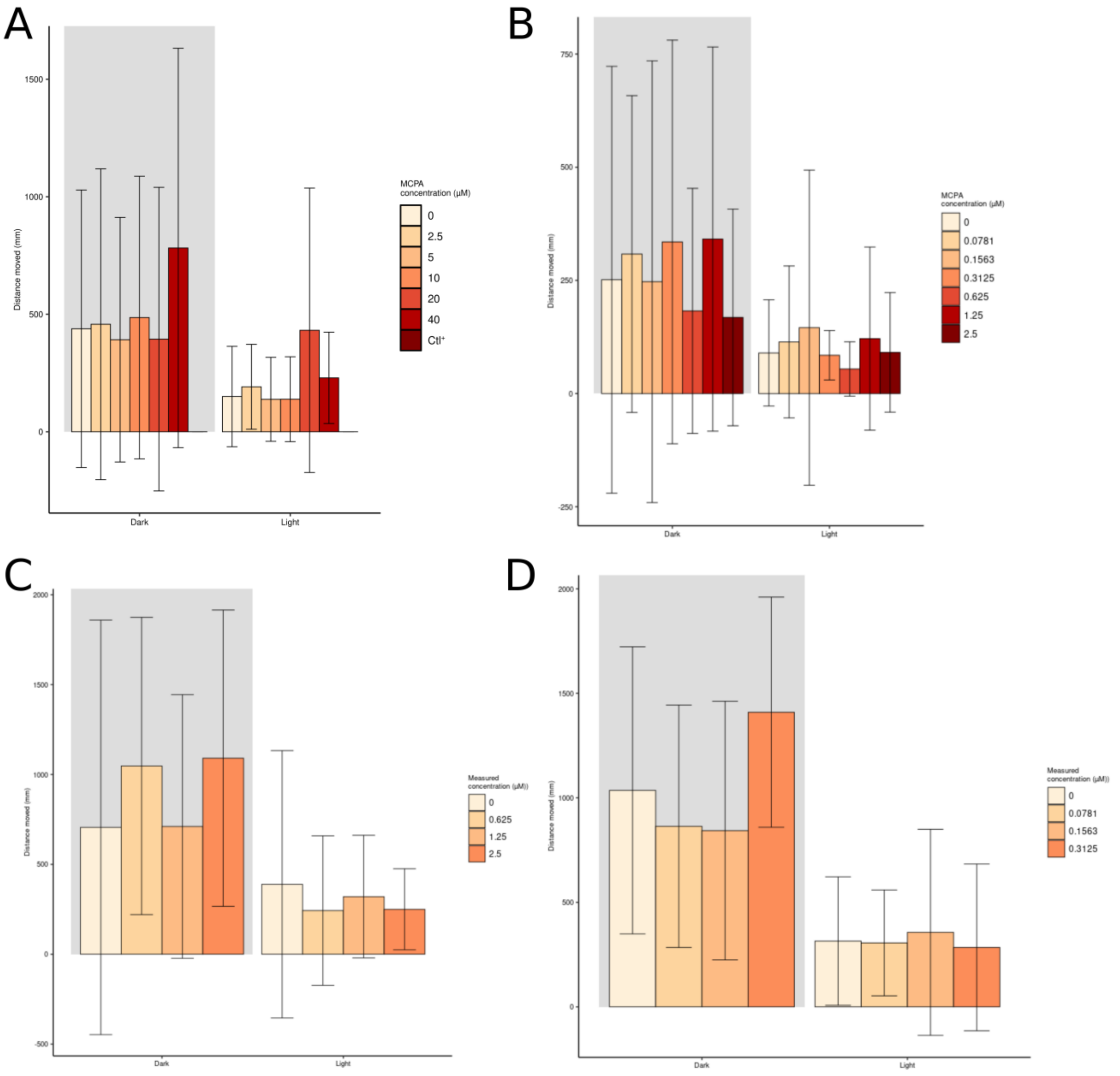

Figure S2. Total swimming distance in four-day post-fertilization zebrafish larvae exposed to various concentrations of methylenecyclopropylacetate for 20 minutes in A and B, for 1 hour in C and 2 hour in D. The sample size was 12 individuals per concentration in A and B, and 24 individuals per concentrationf in C and D No statistical difference between control mean and tested concentrations Dunnett's post hoc test. MCPA: methylenecyclopropylacetate; Ctl+ : 3,4-dichloroaniline.

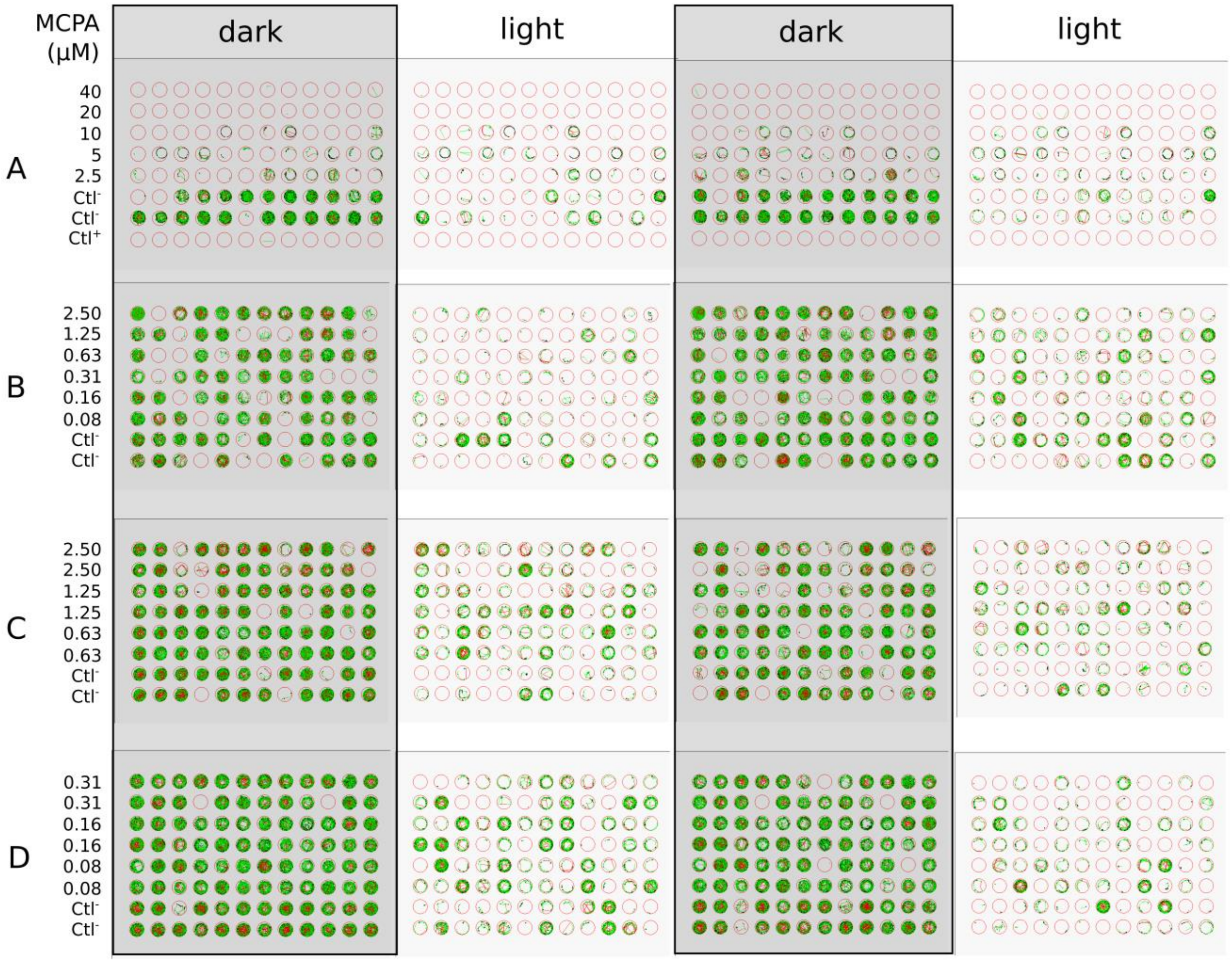

Figure S3. Schematic of zebrafish movement during a 10 min dark and light photocycle, with red lines depicting fast movements, green lines depicting slow movements and black lines depicting inactivity. Zebrafish larvae were exposed to various concentrations of methylenecyclopropylacetate from 72 to 96 hours post-fertilization. MCPA: methylenecyclopropylacetate; Ctl$^+$: 3,4-dichloroaniline. Ctl$^-$: E3 medium

SUPPLEMENTARY TABLE

Test	Toxic	Concentration (µM)							
	HGA	1000	100	10	1	0.1			
	MCPA	4000	400	40	4	0.4			
RFT		1000	100	10	1	0.1			
	MCPrG	3000	300	30	3	0.3			
		5000	500	50	5	0.5			
ZFET +	HGA	200	100	50	25	12.5	6.25	3.13	1.56
24h	MCPA	10	5	2.5	1.25	0.63	0.31	0.16	0.08
Dosage	HGA	100	25	6.25	1.56				
	MCPA	10	1.25	0.07					
Zebrabox	MCPA	40	20	10	5	2.5			
		2.5	1.25	0.625	0.3125	0.1563	0.0781		

Table S1. List of the tested concentrations during the experiments. RFT: Range finding test; ZFET: zebrafish Embryo Acute Toxicity Test;
